# Reproductive concerns and fear of cancer recurrence: a qualitative study of women’s experiences of the perinatal period after cancer

**DOI:** 10.1186/s12884-021-04208-3

**Published:** 2021-10-30

**Authors:** Ruth Naomi Vanstone, Karen Fergus, Noor Niyar N. Ladhani, Ellen Warner

**Affiliations:** 1 York University, 4700 Keele St., Toronto, ON M3J 1P3 Canada; 2 Sunnybrook Health Sciences Centre, 2075 Bayview Ave., Toronto, ON M4N 3M5 Canada

**Keywords:** Cancer survivorship, Perinatal period, Women’s health, Qualitative

## Abstract

**Background:**

Young female cancer survivors are at a disproportionate risk of suffering significant psychological distress following treatment, particularly fears of cancer recurrence (FCR). While previous research has established the robust relationship between FCR and family matters (e.g., family planning and motherhood), there is a paucity of information about how a history of cancer affects women’s psychological functioning throughout the perinatal period. The present investigation sought to better understand women’s experiences of pregnancy and the postpartum period following cancer treatment through a qualitative analysis.

**Methods:**

Ten women participated in a semi-structured, one-on-one interview either over telephone or video conferencing (Zoom). Women were recruited from Sunnybrook Health Sciences Centre in Toronto, as well as through online cancer support platforms, and social media sites. Participants all had a past cancer diagnosis; no active disease; were 45-years of age or younger; currently in the perinatal period; and spoke English fluently. The study employed a grounded theory analysis by which verbatim interview data were analysed using a constant comparison method until data saturation was reached.

**Results:**

The qualitative analysis yielded *I’m So Happy, But Also Terrified*, as the core category, indicative of the duality of emotional experience that characterized the perinatal period for these women. Additionally, four higher-order categories emerged revealing how women go through a process of grief related to potential fertility loss; conditional joy during and after pregnancy due to the lingering weight of cancer; frustration with a lack of resources regarding perinatal health after cancer; and hope as they enter into motherhood.

**Conclusion:**

These results suggest that women in the perinatal period with a history of cancer may be at an increased risk for psychological distress and require additional fertility and reproductive resources both during and after cancer treatment. This research is an important step in further understanding women’s experiences of pregnancy after cancer and may help to inform future research and healthcare practices, in addition to improving perinatal care after cancer.

**Supplementary Information:**

The online version contains supplementary material available at 10.1186/s12884-021-04208-3.

## Background

In 2020, over 19 million new cancer cases were diagnosed globally [1]. Fortunately, due to medical advancements, cure rates have shown a steady increase [2]. Nevertheless, living with a history of cancer often introduces long-term psychosocial challenges including increased rates of depression, anxiety [3–5], fatigue, cognitive impairment, and chronic pain [6]. Furthermore, it is well documented that, although young women comprise a minority of cases, (5.8%) [7] they are at increased risk of experiencing elevated distress [8–10], particularly fear of cancer recurrence (FCR) [4, 5, 10, 11].

FCR is defined as “the fear or worry that the cancer will return or progress in the same organ or in another part of the body” ([12], p. 241). FCR has been shown to be ubiquitous across cancer types [13] and affects a significant proportion of cancer survivors at least to a moderate degree [9]. Clinically significant levels of FCR are often associated with other psychiatric disorders, most commonly anxiety disorders such as panic disorder and generalized anxiety disorder [14]. Unfortunately, the identification and treatment of FCR may be difficult given that it often occurs only in relation to specific situations such as routine follow-up care or the anniversary of diagnosis. Furthermore, FCR may persist for a decade or more after treatment [10, 15, 16].

With respect to young, female cancer survivors, there is evidence that specific periods of life may increase vulnerability to FCR distress. For example, women who are actively engaged in family planning show elevated levels of FCR [17]. One recent study on FCR in breast cancer patients reported that women may worry specifically about the possibility of passing down cancer predisposition genes to their children [18]. In addition, higher levels of FCR have been observed in women with children due to mothers’ concern regarding the emotional impact that a recurrence would have on their family [9]. However, there is a paucity of information detailing how a history of cancer and FCR affect a woman’s experience of pregnancy and the post-partum period.

### Treatment-related fertility concerns

Many young female cancer survivors wish to have biological children, and, in fact, this desire may become more pronounced after treatment [19]. A recent study reported that the chances of becoming pregnant after cancer vary between disease site with some (e.g., bone cancer) showing a reduced rate as compared to others (e.g., non-Hodgkin’s lymphoma) [20]. The vast majority of studies have focused on pregnancy after breast cancer with pregnancy rates as high as 14% [21]. Given the variability of pregnancy outcomes across disease site, it is not surprising that women often worry about whether pregnancy is still possible, and these concerns are not unfounded given that cancer treatments may impede a women’s reproductive ability [22] (e.g., chemotherapy and pelvic radiation therapy may damage healthy oocytes and antiestrogen therapy often used to treat breast cancer disrupts female hormone production) [23]. Additionally, a major concern for women with a history of breast cancer, a hormonally driven tumour, is the safety of subsequent pregnancy given its greatly elevated hormone levels. In some instances, women are given the option to preserve fertility during cancer treatment with gonadotropin-releasing hormone (GnRH) drugs, such as Leuprorelin and Goserelin, which help to prevent ovarian follicles from maturing, thereby protecting them from the effects of radiation and chemotherapy [24]. Other options may include cryopreservation (freezing embryos or eggs) before the onset of cancer treatment to preserve healthy oocytes, ovarian transposition, which moves the ovaries away from the site of radiation, or progesterone therapy, offered specifically for uterine cancer [25]. Despite these options, fertility is often cited as one of the greatest concerns for women of childbearing age both at diagnosis and after treatment [19] but is not often discussed with women before treatment. In one U.S. study of female patients 18-40 years of age, many participants did not recall any discussion regarding fertility options before the beginning of treatment and only 5% were referred to a fertility specialist. This lack of communication was particularly pronounced for women who were single [26].

Although current literature suggests that neither pregnancy [27] nor breastfeeding [28] increases the risk of recurrence, women may still experience heightened psychological distress both when considering becoming pregnant, and during pregnancy. In addition, the majority of studies focus specifically on breast cancer outcomes [21] rather than various disease sites, which may contribute to the uncertainty and distress experienced by cancer survivors.

### Pain, FCR and pregnancy

It is not uncommon for women treated for cancer to be left with some degree of chronic pain due to nerve damage from surgery, radiation and/or chemotherapy and the presence of these sensations, particularly if they occur at the site of the original cancer, often heighten FCR [29]. The physiological changes of pregnancy can exacerbate these symptoms. Furthermore, the breast engorgement and benign breast lumps that are a common occurrence during pregnancy and lactation [30] may be especially worrisome for women with a history of breast cancer [31].

### Perinatal anxiety

Regardless of a history of cancer, the perinatal period (defined as the period from conception up to one-year postpartum) is associated with increased anxiety [32, 33]. Recent research has shown that perinatal anxiety can significantly impact the mental and physical health of both mother and child including fetal distress, preterm birth, increased admission to neonatal ICUs, low birth weight [32], pre-eclampsia, miscarriage, increased postpartum depression [34], and increased physical symptoms [33]. Additionally, pre-existing anxiety and depression, which are more common in women with a history of cancer, put women at increased risk for *pregnancy-specific* anxiety, such as fear of pain during birth and fears about the newborn child’s health, both of which increase the likelihood of adverse outcomes [35].

### Rationale for the present study

Given the high reported prevalence of pregnancy-related anxiety and FCR among female cancer survivors, and the adverse consequences for both mother and child of general perinatal anxiety, it is important to understand more precisely how a history of cancer may further increase women’s psychosocial distress during the perinatal period, a time when women are normally highly vulnerable. By better understanding the perinatal experience of cancer survivors, earlier identification of those women who need intervention and special support may be possible with the ultimate aim of improving the physical and mental health outcomes of mother and baby.

The aim of the present study was to understand women’s lived experiences of the perinatal period after having received treatment for cancer and the ways in which women coped, both physically and emotionally, during their pregnancies and into the postpartum period. Additionally, we were interested in how women dealt with navigating a healthcare system with sparse information related to their unique maternal trajectories. Finally, we were interested in how women perceived their healthcare provider support in the transition from cancer treatment into pregnancy, and other sources of support that they found helpful throughout the process. Given the limited amount of extant research examining this phenomenon a qualitative approach (patient interviews) was deemed most suitable.

## Method

### Participants

A total of 10 women participated in the study. Inclusion criteria were as follows (1) past cancer diagnosis (across cancer type); (2) no active disease; (3) 45-years of age or younger; (4) currently in the perinatal period; and (4) the ability to read and speak English fluently. Although recruitment was world-wide (as per below), all but one participant resided in Canada. All women were in a heterosexual relationship and living with their partner. Only one of the ten women had children prior to her cancer diagnosis. Four women were currently pregnant, and six women were between 2.5 and 10 months postpartum. Of the four pregnant women, none had any children. No conflicts of interest were identified. In total, 14 women contacted R.V. to participate in the study. Nine women were directed from online cancer support sites, eight of whom met inclusion criteria, and ultimately participated in the study. The other five women were approached by their health care providers at Sunnybrook hospital. All five women contacted R.V. initially. One woman did not meet inclusion criteria, and the other two did not follow-up to complete the interview. Participants were primarily recruited (80%) through advertisements placed on online cancer support websites across North America and Europe or social media subsites dedicated to cancer survivors (e.g., Reddit), the remaining participants were recruited through their primary care providers (oncology) at Sunnybrook hospital. Interested individuals contacted the study coordinator by email or phone to confirm that they met inclusion criteria. Once criteria were confirmed, each participant was sent an informed consent form via Qualtrics and, upon informed consent, an interview was scheduled. For a summary of demographic, disease-, and pregnancy-related demographics, refer to Table 1.

**Table 1:** Sample characteristics (*N =* 10)

ID	Age	Ethnic background	Education	Cancer type	Cancer treatment	Fertility preservation	Method of conception	Method of birth	Time between diagnosis and pregnancy	Current trimester or age of child
001	40	White	Graduate degree	Non-Hodgkin’s lymphoma	Chemotherapy	Cryopreservation	Spontaneous	Emergency cesarian section	5 yrs	5.5 mos
002	31	White	Professional degree	Leukemia	Chemotherapy	None	Spontaneous	N/A	1 yr 2 mos	2nd
003	35	Middle Eastern	Bachelor’s degree	Breast	Surgery Chemotherapy	Not given option	Spontaneous	N/A	5 yrs	2nd
006	37	White	Bachelor’s degree	Non-Hodgkin’s lymphoma	Chemotherapy	None	Spontaneous	Vaginal	2 yrs	2 mos
007	31	Mixed race	Graduate degree	Non-Hodgkin’s lymphoma	Chemotherapy Radiation	None	Spontaneous	N/A	9 yrs	2nd
010	41	East Asian	Graduate degree	Breast	Surgery Chemotherapy Herceptin	Cryopreservation	Spontaneous	Vaginal	1.5 yrs	2 mos
012	27	South Asian	Bachelor’s degree	Breast	Chemotherapy Radiation	GnRH	Spontaneous	N/A	1 yr	2nd
013	38	White	Graduate degree	Breast	Surgery	None	Spontaneous	Scheduled cesarian section	10 mos	10 mos
014	37	White	Bachelor’s degree	Breast	Surgery Chemotherapy Radiation Hormone therapy Herceptin	Cryopreservation	Spontaneous	Vaginal	4 yrs	2.5 mos
015	35	White	Bachelor’s degree	Breast	Surgery Chemotherapy Radiation Herceptin	Cryopreservation GnRH	Artificial reproductive technology	Cesarian section	3 yrs	10 mos

### Procedures

The study protocol was reviewed and approved by the Research Ethics Board (REB) of the Sunnybrook Health Sciences Centre (REB #344), and the York University Human Participants Committee prior to participant recruitment. Informed consent was obtained from all participants before any data were collected.

Data collection consisted of one semi-structured interview with each participant, all of which were approximately 60 min in length. All interviews were conducted by R.V., a female master’s student in clinical psychology at the time of the interviews. R.V. had previous training in both clinical and qualitative interviewing. R.V. had no previous relationship with any of the participants. Although R.V. did not explicitly state her personal goals for the research, she shared with participants her own interest in the topic at the end of the interviews, if they asked. Specifically, the interviews focused on women’s emotional experiences and coping efforts in relation to the various challenges they faced during both cancer and pregnancy and (where applicable) postpartum. In addition, two women who were outside the perinatal period but did have a history of cancer were consulted in the development of the interview guide and shared suggestions for possible questions that may illuminate women’s experiences (see Additional file 1 for the full semi-structured interview).

All interviews were conducted via telephone, except one held via Zoom version 5.0.2, a secure, online videoconferencing platform. Both R.V. and each participant participated in the interview from their home, with no others present. Consistent with qualitative methodology, data collection and analysis were conducted simultaneously thus allowing the opportunity to add questions to the interview guide as necessary based on themes that emerged during the interviews.

### Analysis

All interviews were audio recorded and transcribed verbatim. The text was analyzed by the first author (R.V.) in ongoing consultation and discussion with K.F., a clinical psychologist and qualitative researcher using a grounded theory approach, initially proposed by Glaser & Straus [36], and subsequently adapted for use in psychology by Rennie, Philips, & Quartaro [37]. We chose a grounded analysis for this particular research due to the dearth of studies regarding women’s experiences of the perinatal period after cancer. The grounded theory method allows for the data to guide the development of an emergent theory by using an inductive approach during the initial phases of coding interview text, and subsequently, a deductive approach wherein the emerging categories are analyzed together as a whole data set [38]. Given there is a paucity of literature regarding the current topic, the grounded theory approach allows a rich and nuanced examination of the topic. In addition, this method helps to ensure that the analysis is reflective of the data, and thus the emergent theory is generated rather than verified by predetermined theoretical concepts [36]. Each interview was divided into excerpts of text, referred to as ‘meaning units’ (MUs) [39] - each of which represented a single theme or concept. Each MU was then assigned a category label representative of the essential idea or ideas contained within it. The constant comparison method [37] was applied such that each new MU was compared to existing categories. If none of the codes adequately captured the theme or concept being expressed in the MU, a new category was created. In some instances, existing categories were re-named in order to more adequately capture and integrate the new data. This process continued until no new categories emerged from the data, thus reaching saturation. Given this was a convenience rather than theoretically derived sample [37], the type of categorical saturation achieved in this context was bounded by the fact that this was an educated relatively socio-economically advantaged sample of women with access to universal health care. Categorical saturation was reached after nine interviews, although a tenth interview was conducted to ensure we had passed the point of saturation. This is consistent with literature stating that as many as 10 interviews may be necessary to uncover more nuanced codes in the data if no new codes emerge from three consecutive interviews [40].

Once the transcripts were coded, a hierarchical structure was created by grouping together categories with a shared meaning element. Lower-order categories were organized such that each grouping of codes was indicative of a newly created, higher-order category, guided by the broader research question of whether and how women with a cancer history experience psychological distress during the perinatal period as a result of this history. This process continued until one ‘core’ category was constructed to capture the phenomenon of interest, representing the full data set.

## Results

The grounded theory analysis resulted in a total of 43 lower-order codes, which were subsequently organized into nine higher-order categories. These nine higher-order categories represent the defining features of the four main categories, from which a core category emerged, *“I’m So Happy, but Also Terrified”* representing women’s experience of the perinatal period after cancer. The core category was reflective of the finding that women in the perinatal period experience a concurrent duality of emotions, uniquely influenced by their history of cancer.

**Fig. 1:**
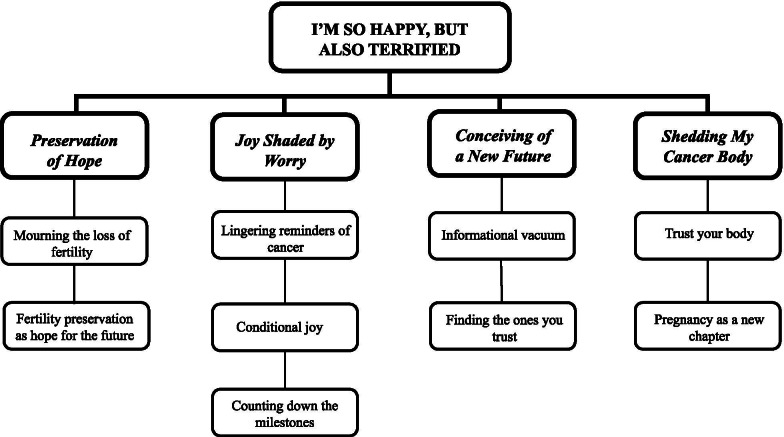
Higher-order categories and their defining properties

Despite individual differences, every participant’s experience of pregnancy after cancer included at least one element of the following (representing the four main categories): (1) *Preservation of Hope*, in which participants expressed a narrative of grieving the possible loss of fertility but finding hope in the measures taken for fertility preservation before or during cancer treatment; (2) *Joy Shaded by Worry*, wherein participants expressed conditional joy while pregnant and, in some cases, into the postpartum period; unable to embrace their excitement due to worries of recurrence and the health of their child; (3) *Conceiving of a New Future*, which represented women’s frustration with the lack of information regarding fertility options during and after cancer treatment, and the steps they later took to ensure they had the best possible healthcare team throughout their pregnancy journey; and (4) *Shedding My Cancer Body*, in which women expressed that, although cancer would always be a part of their narrative, pregnancy seemed to define a boundary between the two experiences of cancer and motherhood. These four higher-order categories are summarized in Fig. 1 below. The following presentation of the findings include quotations, which have been chosen to illustrate the categories, and labelled in brackets with the participant’s unique ID number, and age.

### Preservation of hope

#### Mourning the possible loss of fertility

Every participant (*n* = 10) expressed that the potential loss of fertility was salient to them after receiving their cancer diagnosis. As one woman said, “I think that was one of the first things that came to mind at that point, ‘Would I ever be able to have kids?’” (P012, 29). It was extremely difficult to try and process that the future they had planned for, which included having children, might not be possible, especially as they were simultaneously processing their cancer diagnosis. The majority of women (*n* = 8) expressed that the idea of the potential loss of fertility was devastating to consider and, in some cases, even more devastating than the cancer diagnosis. As one woman said:

I was I think grieving this possibility of [infertility]…That was harder for me. I was sad that maybe we won’t be able to have kids, or we won’t for five years or you know, we don’t know if it will even be possible… (P013, 38)

This loss was particularly difficult to come to terms with as women felt that their diagnosis had derailed their plans for the future. Women’s distress was compounded by the uncertainty of the effect of treatment on fertility. The majority of women (*n* = 9) struggled with the fact that they were unsure of whether treatment would result in infertility and found it difficult to process the ambiguity of a potential loss. This upset was compounded, for some women (*n* = 4) by the fact that fertility took a back burner to cancer treatment. As one woman stated, “I guess there’s so many things going on at the time of diagnosis, like coordinating a surgeon and oncologist…that maybe it doesn’t seem like as high of a priority, or it gets forgotten because of that (P015, 35). The ‘forgotten’ loss of fertility was perceived, for some women, as another point of distress in addition to the diagnosis.

#### Fertility preservation as hope for the future

Although the idea of fertility loss was distressing for women, it was juxtaposed with the hope offered by the opportunity for fertility preservation. Nine women had the opportunity to speak with a fertility specialist. However, only five women recalled that their doctors brought up the subject of fertility preservation without prompting. One participant stated that, because she was single at the time of diagnosis, she was told that fertility preservation was not an available option before treatment. The majority of women (*n* = 7) expressed that they viewed fertility preservation as providing a sense of control throughout their cancer treatment as well as hope for the future. Other women (*n* = 4) said that merely going to see a fertility specialist and better understanding their options greatly helped their coping. One woman said of speaking about the possibility of having children in the future, “It is something that I always wanted to do and so knowing that there were options in that regard helped me to just feel as though I had a bit of control over that aspect of that treatment” (P014, 37). Another woman said:

I feel grateful that I had the opportunity to end up freezing some eggs. I didn’t end up using them but it was actually quite helpful for me in terms of coping…to know that cancer wasn’t taking that away from me, you know, it was still an option. (P001, 40)

### Joy shaded by worry

#### Lingering reminders of cancer

The majority of participants (*n* = 9) experienced cancer triggers throughout their pregnancy, which began for some women as soon as they found out they were pregnant up until giving birth. One woman stated that going for ultrasounds was distressing due to their negative association with breast imaging. Other women (*n* = 2) had negative thoughts about giving birth in the hospital as hospitals elicited memories of treatment. One woman said, of touring the hospital in preparation for giving birth, “I remember on the hospital tour actually just bursting out - I had to leave and was just in tears, I was like ‘I can’t do this I don’t want to have a hospital birth’... they’re [hospitals] filled with fear” (P001, 40). As one woman said, “You do carry with you, kind of like medical trauma…you kind of get into a mindset that you’re always going to receive terrible medical news” (P002, 31). Women felt that cancer would always be a part of their narrative that would carry forward into other experiences and colouring other aspects of life.

#### Conditional joy

The idea of conditional joy was endorsed by the majority of women (*n* = 9) when speaking about their experience of pregnancy and, in some cases, after giving birth. On the one hand, pregnancy elicited overwhelming joy for the majority of women (*n* = 9) all of whom had thought at some point throughout their cancer journey that pregnancy may not have been a possibility. However, the joy was coupled with various fears, engendered by the experience of cancer diagnosis and treatment. For example, some women (*n* = 4) worried about the lingering effect that chemotherapy may have had on their bodies, such as the possible damage to their eggs and the safety of the fetus in a post chemotherapy body. As one woman articulated:

For me it was like ‘Well what if… the quality of my eggs has been compromised because of chemo? So, what if there's potential for, genetic diseases?’ All of these terrible things were kind of my initial thoughts. So it was that happiness of like, ‘Oh my god it happened and like we really weren't expecting it to, but then that constant fear of something will go wrong. (P007, 31)

Others were explicitly worried about their cancer returning, “You’re so overjoyed about this baby, but then you’re terrified that your cancer is going to come back” (P006, 37), while others mentioned their worries about passing on cancer genes to their children (*n* = 6). These women expressed that they wanted their children to be safe and healthy, to have a ‘fighting chance’, and said they had feelings of guilt, or selfishness for wanting to conceive and raise children, knowing that their children may, at some point in their lives, be affected by cancer.

Their cautious happiness was always fueled by their history of cancer and represents a complexity of experience that is very unique to these individuals. As one woman said of becoming a mother, “Actually, unfortunately I had to mentally process this, though it gets easier … the fear never completely goes away” (P014, 37). By the same token, the worry about cancer returning did not overshadow women’s joy of being pregnant. Rather, it rendered the joy conditional and subject to moments of worry specific to each woman’s cancer history.

#### Counting down the milestones

Pregnancy is necessarily coupled with various milestones, that women looked toward for reassurance of the health of their child. As evidenced above, women contend with many worries during pregnancy and they tended to assuage these worries by seeking healthcare provider reassurance throughout their pregnancy (*n* = 6) and taking comfort in moving safely from one milestone to the next (*n* = 6). As one woman put it, “Once you clear certain hurdles, you feel a lot better” (P002, 31). In a sense, women felt as if they were holding their breath, hoping to make it safely to the next important milestone.

Participants (*n* = 6) also expressed that the first trimester was especially challenging and one in which they felt they needed extra reassurance that both they and their baby were healthy and safe. This increased reassurance-seeking appeared linked to the experience of having had cancer, which increased worry for their baby’s health. Several women (*n* = 6), for example, indicated that once they made it past the first trimester, in which the rate of miscarriage is highest, that they felt as if they could relax more into their pregnancy. One woman described that knowing the rate of risk for various complications such as miscarriage and genetic mutations, and then passing those milestones acted as a way of gaining control over something that was ostensibly out of her grasp.

There is an overall tendency to take comfort in the milestones that pass (e.g., after the first trimester rate of miscarriage decreases), to be able to exhale for a period of time. Although some worry in pregnancy is likely normal, women with a history of cancer seemed especially anxious about reaching these milestones, given the dearth of information on the impact of cancer treatment on pregnancy. This sentiment was reflected in the relief that women felt when being reassured that they had no health complications as their pregnancies advanced.

### Conceiving of a new future

#### Informational vacuum

Although each participant was able to conceive after cancer, all expressed some dissatisfaction with the limited amount of fertility information they had access to both during and after treatment. They expressed frustration with this informational void. Some (*n* = 3) identified that there was a general lack of information for women their age. One woman stated, “A lot of the information… out there were for women who had cancer later on in life after they’ve had kids…That’s all the information I could find” (P012, 29). This frustration was compounded by the fact that, as many women (*n* = 5) divulged, even their healthcare providers were not able to provide information - mostly due to lack of knowledge. In speaking with her fertility specialist, one woman commented, “I remember asking about…chemo and how that would affect fertility and him saying, ‘You know, I don’t really know’” (P001, 40). As a result of the lack of information forthcoming from health care providers, participants expressed having to draw on their own resources to gain a sense of understanding about issues regarding pregnancy after cancer. Specific concerns expressed by women in the study were whether artificial reproductive technologies (ART) would fuel their tumour growth (*n* = 1), the length of time they should wait after the end of treatment to become pregnant (*n* = 2), and the effect of ceasing hormone therapy (*n* = 1).

Most of the women (*n* = 9) commented on how the lack of information about fertility, in particular, compounded the stress of cancer treatment. These feelings manifested both before and after treatment, either when trying to understand their chances of conceiving after cancer, or if successful, the safety of pregnancy for themselves and the newborn. At some stage, whether seeking information about fertility preservation before cancer treatment, or in thinking about conception after treatment, every woman felt that there was uncertainty and often very little, if any, information they could find as if they were feeling their way in the dark for a light switch that does not exist.

#### Finding the ones you trust

The lack of information on fertility that characterized their experience during cancer treatment seemed to spark a sense of self-advocacy when entering into the perinatal period as women became savvier in navigating the healthcare system and garnering the support they needed. The majority of participants (*n* = 8) spoke about the importance of finding perinatal healthcare providers (such as obstetrician/gynecologists and family doctors) with whom they felt comfortable. Some women (*n* = 4) spoke of ‘shopping around’ until they found those whom they felt would give them the best quality of care.

Three women also recalled the importance of self-advocacy throughout the perinatal period. As one woman said of the medical system, “It doesn’t have all the answers, so you do need to be your own advocate” (P001, 40). Another woman attributed her ability to advocate for herself throughout the perinatal period to her experience with cancer, as she was able to apply what she learned as a cancer patient to her experience in the perinatal period:

I think that there is something that happens to at least a percentage if not all people who go through treatment, I don’t want to say become an expert at things, but definitely you become a bit more savvy and you learn about what makes you feel more comfortable when you are being taken care of. (P014, 37)

Working with a trusted healthcare provider was integral to helping women navigate the unknowns when trying to become pregnant after cancer.

### Shedding my cancer body

#### Trust your body

Nearly all participants felt that there was a clear distinction between their ‘cancer body’ and their ‘pregnant body’ (*n* = 8), specifically when relating to bodily sensations during pregnancy. For example, one woman stated that, although some symptoms of pregnancy were similar to those of chemotherapy, she was able to distinguish readily between the two and felt no added worry. In fact, two women said that, albeit unpleasant, symptoms of pregnancy such as morning sickness and back pain, were reminders of their ability to become pregnant and were therefore more associated with feelings of joy rather than worry.

Two women expressed that their body gave them “clues” that they were ready and able to conceive. One woman said that, although she experienced morning sickness and back pain during pregnancy, these symptoms were perceived as positive signs of a healthy pregnancy. Similarly, another woman stated that she was optimistic to be still menstruating (after having been through chemotherapy), and felt that it may be a sign of fertility, “I was still getting my periods, so I thought maybe it’s not such a bad thing, as in, it’s not a done deal, as in, ‘It’s not as if I’ll never be able to have children’” (P003, 35). Interestingly, most women (*n* = 8) could easily distinguish ‘normal’ pregnancy pains from cancer-related discomfort or pain, despite some symptoms being similar such as nausea from chemotherapy versus morning sickness.

#### Pregnancy as a new chapter

Regardless of the undeniable burden that a history of cancer brings, all of the participants (*n* = 10) expressed how fortunate they were to have become pregnant after cancer. For participants, pregnancy felt like a ‘win against all odds’ or as one participant strikingly put it, that she was “the luckiest of the unluckiest” (P002, 31). Some of the participants (*n* = 4) also expressed that cancer helped them gain perspective about what their bodies were capable of. As one woman said:

I thought ‘childbirth looks excruciating and it looks so painful and difficult’. And I probably feared it but …I was looking forward to meeting my baby and having a baby, and I was just so grateful that I could be pregnant. (P010, 41)

There was a sense that getting through cancer prepared the women for other difficult experiences they might encounter throughout the perinatal period.

Not only did the physical feats associated with pregnancy seem easier after cancer, but some women (*n* = 2) also reflected on how the experience of cancer offered some perspective as they embarked on the journey into motherhood. For example, one woman said, “I think as women who’ve experienced cancer you know, you develop so many skills and tools to navigate the unknown” (P001, 40).

Some women (*n* = 4) viewed pregnancy as a protective barrier between themselves and the cancer and expressed gratitude for the ability to conceive. They were able to ‘look back’ on the cancer experience and draw a boundary between their perinatal selves and their cancer selves. As one woman reflected, “I’m looking at it from the other end now, like, that I just recently had a baby and I’m just incredibly grateful that- that I could” (P010, 41). Another woman succinctly stated, “I’m pregnant [laughter]… I’m more optimistic about the future” (P003, 35).

Although cancer is never forgotten, pregnancy represents a hopeful way forward; a way to delineate the dark journey that was cancer, from the possibility of a bright future ahead:

Remembering when I thought I couldn't have kids or just remembering really difficult aspects of going through the chemo…I would say that I do feel almost more distanced from it now that I'm pregnant and going through this…. chapter… I will view being pregnant as the ultimate symbol of being done with it. (P002, 31)

## Discussion

The present study was conducted in an attempt to better understand how a history of cancer affects women’s experiences of the perinatal period. The diagnosis and treatment of cancer can be an exceptionally distressing experience that may continue to be psychologically distressing [3–5, 8–10] long after the end of treatment. FCR is consistently reported as one of the greatest challenges for cancer survivors [10, 11, 15] and is particularly pronounced in young women [9, 41]. The current study builds on prior evidence that FCR may be related to motherhood and sheds light on the experience of the transition from cancer patient to women in the perinatal period, their specific worries during pregnancy and postpartum, and how their history of cancer may help to inform them of how best to cope with challenges during the perinatal period.

The core category that emerged from our analysis, *I’m So Happy, but Also Terrified*, illustrates the complexity of women’s experience and the duality of emotional responses that women exhibit during the perinatal period. Our results suggest that women, while excited by pregnancy, also endure long-lasting effects of cancer such as worries about the safety of fertility preservation (e.g., increase in hormones necessary for preservation that may fuel cancer growth in hormone receptor positive women) and pregnancy, and the experience of medical triggers. We found that women sought to cope with the consequences of their cancer history by increasing their own knowledge of perinatal health and seeking support from health care providers whose knowledge they trusted.

Previous research suggests that fertility preservation is of utmost importance to women of childbearing age who are diagnosed with cancer and who want to have children [28]. Women report an increase in depression and psychological distress when they perceive a lack of information regarding fertility preservation and in relation to fertility loss [42]. Conversely, when women are provided with increased fertility resources and options for preservation, they report increased levels of hope and a greater sense of control throughout their cancer treatment [43, 44]. Our findings are consistent with this trend, as each participant revealed that, either at diagnosis or early into treatment, fertility preservation was a major concern and, in fact, that the choice to preserve fertility helped to alleviate their distress during treatment.

Interestingly, participants were likely to deal with such uncertainty through action and information seeking, which may have positive implications for coping and psychological outcomes. Studies have demonstrated that Uncertainty Tolerance (UT) may determine psychological outcomes, both negative and positive, and is especially salient in relation to life-threatening illnesses such as cancer given the ambiguity of future outcomes [45, 46]. Negative cognitive and emotional appraisals of uncertainty may elicit feelings of vulnerability, worry, fear, and anxiety [46]. All but one participant took action to better inform themselves of the risks of cancer treatment on fertility either by speaking to a fertility specialist and/or by accessing online information about fertility preservation. Additionally, seven of the 10 participants took further action to preserve fertility through egg freezing (*n* = 5) or taking temporary protective drugs throughout cancer treatment which help to protect ovaries (*n* = 2). Participants expressed that taking action to preserve their fertility was integral to their coping throughout cancer.

Women’s experience of cancer triggers during the perinatal period is also consistent with extant literature, which suggests that cancer survivors often experience heightened FCR in specific medical situations such as during appointments with healthcare providers, examinations, and other procedures, which can activate negative memories of cancer treatment [16, 47]. Similar findings are reported in the FCR literature which documents that while up to 92% of cancer survivors report hopes of having children post-treatment, they worry about the effect of cancer treatment on subsequent pregnancies and report heightened levels of fear when thinking ahead to parenthood [48]. Given that past literature also reports heightened FCR for women who are already mothers [9, 41], it is not surprising that women would worry about these issues during the perinatal period as well.

The one unanticipated finding, based on previous understanding of the interrelationship between FCR and hypervigilance [10], was the reduction of concern around bodily symptoms reported by this sample of women. While more research is needed, this finding suggests that pain and physiological experiences that may normally trigger FCR may not be at play to as great a degree for a woman while pregnant because the more common symptoms of pregnancy (e.g., nausea, back pain) appear to be interpreted as such, despite being reminiscent of treatment side effects. Instead, they are interpreted as normal physiological changes due to pregnancy and may, in fact suggest that pregnancy acts as a protective period for women with a history of cancer.

Another major theme that emerged from our analysis was participants’ increased knowledge of the healthcare system after cancer, which sparked, for some, actions related to self-advocacy into the perinatal period. All participants cited a lack of information regarding fertility preservation options before cancer treatments, as well as a lack of information about the risks of pregnancy after cancer. This dearth of pregnancy-related information coupled with the known fertility risks posed by cancer treatment were a pronounced source of distress and frustration for all participants. This finding is not unique. Despite ongoing research regarding both fertility preservation throughout cancer treatment, and the safety of pregnancy after treatment, women continue to report a paucity of information [49]. Research shows that access to informational resources is integral to cancer patients’ and survivors’ well-being and psychological health. Specifically, information that is clearly communicated and well understood [50, 51].

Interestingly, this lack of information seemed to spark women’s proactivity in searching for informational resources. Lober & Flowers [52] observed that, with the increase in available health technology, younger patient cohorts have become more engaged with their own health care. As a consequence, younger cohorts have become more empowered to navigate these technologies in order to assume a more active role in their care [52]. This phenomenon is evident with baby boomers, and now millennials who have become more knowledgeable and engaged consumers and who, generally, seek out patient-centered care [52]. This increase in engagement was noted in a majority of our participants, especially in the transition from cancer patient to perinatal patient.

While it is difficult to provide general information for patients given the variability of diagnosis and treatment, the women in our study reported that increased contact with their trusted health care providers was integral to their coping with pregnancy and into the postpartum period. This not only included oncologists, Obstetrician/Gynecologists, and family doctors, but also mental health professionals throughout the perinatal period.

The importance of personal social support is well established in the cancer [53] and pregnancy [54] literature and is shown to positively influence coping. While women in our study each mentioned the importance of partner and family support throughout the perinatal period, they indicated that their relationship with their healthcare provider was paramount to their coping as they were women’s primary source of information. This finding supports work Meggiolaro et al. [55] who found that patients who perceived their healthcare provider to be disengaged and unsupportive reported higher levels of distress and hopelessness. A review conducted by Arora [56] found that positive patient-physician communication in cancer populations is indicative of patient well-being and patients who view their healthcare provider as supportive and knowledgeable show better health outcomes. Our findings reflect this literature and, perhaps, add the nuance that women who have been through cancer are better positioned to self-advocate for increased healthcare provider support during the perinatal period due to their increased awareness of their own needs when in care.

Finally, participants in our study reflected on the fact that, while cancer would never be forgotten, pregnancy represented a new chapter in their lives. Each woman stated that she was more appreciative of her ability to conceive due to the fact that she had been through cancer treatment which jeopardized this very capacity. Taylor and Lobel [57] introduced the notion of downward social comparison - that is, comparing oneself with others who are perceived to be worse off - as a form of coping with life-threatening illnesses. They identified that cancer patients in particular often unconsciously use ‘downward comparison’ as a strategy to cope with threatening aspects of cancer. Similarly, patients used ‘upward’ social comparison techniques to derive hope. Though we did not explicitly explore whether our participants compared themselves to others, we found that they seemed to rely upon a form of *internal* downward comparison, by which they compared their pregnant selves to their prior cancer selves, as a way of feeling more secure about the future. Again, in this way, pregnancy may act as a protective factor as women are able to delineate these two experiences; seeing themselves as having distanced from cancer, they were more able to integrate an optimistic narrative, buffering them from the potential threat of their cancer history.

### Limitations

An important limitation is the lack of diversity in our sample as all women were college educated, and the majority were White. Additionally, our sample was a convenience sample of only four pregnant and six post-partum women. Although saturation was achieved with this sample of 10 women, we acknowledge that nuances may have been missed and that if, for example, our subsample of pregnant and postpartum women were more ethnically diverse, or residing in non-Westernized countries, other themes might have emerged. Thus, the saturation acquired in this study is restricted to the demographic parameters of this particular sample. However, given the fact that saturation was reached in the present analysis gives confidence that, for women of similar sociocultural background, comparable findings would be observed. However, it is important to note that the experiences of women in this study may not be representative of a broader population. Additional research in this area would be helpful in order to demonstrate whether similar results extend to women with a cancer history of differing ethnic and socio-economic backgrounds. Indeed, it is possible that because our participants were highly educated, they were actively seeking information, which impacted their emotional well-being and levels of distress. Finally, the majority of women had access to various health care services, including fertility and mental health services. Again, given the homogeneity of this sample, the results may not resonate with the broader population of perinatal women with a history of cancer. Increasing demographic variability in future studies may result in different findings, thus allowing for a more nuanced understanding of this phenomenon possibly leading to an improved standard of care for this population.

## Conclusions and practice implications

To our knowledge, the present study was the first to examine the psychological effects of cancer on women’s experiences of the perinatal period. The paucity of research regarding how a history of cancer effects the perinatal period represents a significant void in understanding women’s needs after cancer. Thus, the aim of the present study was to shed light upon women’s experiences during this unique time in their lives in order to better understand women’s health and inform future care.

We found that women experience a duality of emotions during the perinatal period that is largely fueled by their history of cancer, encapsulated by the expression, *I’m So Happy, but Also Terrified*. While cancer may always colour other medical experience, women reflected that the perinatal period, particularly pregnancy, represented a comforting delineation between their cancer experience and their future, indicating that pregnancy may act as emotionally protective for women after cancer. Future studies are necessary to determine the protective impact of pregnancy on FCR and other forms of perinatal distress elicited by a history of cancer.

Future studies could look at comorbidities for women who may be more prone to anxiety during the perinatal period and after a history of cancer. This may influence women’s coping during pregnancy in relation to FCR. In general, the psychological effects of cancer during the perinatal period are not well understood and more research is needed in order to better support women during this vulnerable time. Longitudinal research, capturing individual women’s experiences throughout the perinatal period would also be useful in understanding whether certain timepoints are subject to increased distress and vulnerability (e.g., first trimester as most distressing for the majority of women given the increased rate of miscarriage after chemotherapy) – thereby justifying increased informational and emotional supports during this time.

In addition to guiding future research, this study may also shed light on important practice considerations for health care professionals caring for women in the perinatal period who have a history of cancer. Consistent with existing, primarily quantitative literature, the current study’s qualitative findings suggest that women require additional information regarding fertility outcomes both during and after cancer treatment, as well as specialized healthcare provider support. The participants in this study specifically identified that they were most comfortable with healthcare providers who were not only understanding and sympathetic to their concerns, but those who offered informed recommendations, or were willing to work with their patients to find pertinent information to enable more informed decisions. Therefore, as new research emerges, it will be important to continue to offer up-to-date information not only to patients, but also to have increased communication amongst providers and multidisciplinary teams to ensure consistent recommendations and guidelines for best practice.

## Supplementary Information

**Additional file 1.** Semi Structured Interview Guide.

## Data Availability

The datasets generated and/or analysed during the current study are not publicly available due to the depth of disclosure and deeply personal nature of the interview content, and that participants did not provide informed consent to share the raw qualitative text in-full. However, deidentified datasets are available from the corresponding author on reasonable request.
