# Supplementary material for: Reproductive concerns and fear of cancer recurrence: a qualitative study of women’s experiences of the perinatal period after cancer
Source: BMC Pregnancy Childbirth. 2021 Oct 30;21:738. doi: 10.1186/s12884-021-04208-3 (PMC8556905; doi:10.1186/s12884-021-04208-3)
Supplement: Supplementary file 1 — Additional file 1. Semi Structured Interview Guide. [file 12884_2021_4208_MOESM1_ESM.docx]

**Pregnancy after Cancer**

**Semi Structured Interview Guide**

Preamble: Thanks so much for agreeing to speak with me today. And just to remind you, this interview should take approximately 45 minutes and is being audio recorded. As you read in the Informed Consent Form, you should feel free to speak about your experiences only to the extent you feel comfortable, and you also have the option to decline answering any question if that is your preference. Do you have any questions for me before we begin?

I would like to begin by asking you some demographic questions if that’s ok?

1. Currently place of residence (city/country)
2. Date of birth
3. Self-identified ethnicity
4. Education level
5. Current marital status
6. Previous children
7. Sex and age of children
8. Current living situation
9. Birth complications

Now I would like to ask you some questions about your history with cancer if that is ok?

1. Can you tell me a little bit about your cancer diagnosis and treatment? What type of cancer were you diagnosed with? What stage? When was it?
2. In hindsight, how do you feel you coped with the illness? What were some of the most challenging aspects for you? What did you find helpful?
3. Do you recall considering the possibility of pregnancy (or having more children if applicable) during your illness?
4. How was the process of discussing the possibility of pregnancy after treatment had ended? Who did you discuss it with? What kind of resources did you have access to?
5. How has your experience of pregnancy been thus far? (if pregnant)
6. What was your most recent experience of pregnancy like? (if postpartum) (Inquire also re: breast feeding)
7. Can you talk a bit about your supports throughout the pregnancy? (Partner, HCP)
8. How have you been feeling physically over the course of your pregnancy (or since you gave birth)?
9. How have you been feeling emotionally over the course of your pregnancy (or since you gave birth)? (Inquire also re: changes)
10. Were there any periods you found more difficult than any others?
11. How did you find this pregnancy different from past pregnancies (if relevant to the individual)
12. How do you think your experience of cancer has affected your pregnancy (if at all)? (inquire on an emotional level and physical)
13. What was your experience of dealing with the health care system? What was the support like?
14. How has your experience changed since the onset of COVID?
15. Are you interested in filling out the online questionnaires?
